# Feeder-Free Expanded and Cryopreserved NK Cells Retain Antibody-Dependent Cell Cytotoxicity against HER2-Positive Tumors

**DOI:** 10.4014/jmb.2507.07016

**Published:** 2025-10-29

**Authors:** Injee Lee, Suwoo Kim, Chaeyeon Jin, KyuBum Kwack, Youngseok Baek

**Affiliations:** 1 Department of Biomaterials Engineering, School of Medicine and Biomedical Science, College of Life Science, CHA University, Seongnam 13488, Republic of Korea; 2 R&D Division, CHA BIOTECH, Seongnam 13488, Republic of Korea

**Keywords:** ADCC, NK cells, cellular immunotherapy, cryopreservation, feeder-free expansion

## Abstract

CAR-T, TIL, and TCR-based T cell immunotherapies have revolutionized cancer treatment; however, their clinical application remains limited by severe adverse effects such as CRS, ICANS, and HLH, as well as the risk of GvHD in the allogeneic setting. In contrast, NK cell-based therapies have emerged as a safer alternative with minimal adverse effects and can be developed from diverse cellular sources. Nevertheless, clinical translation of NK cell therapies is challenged by difficulties in large-scale expansion, donor variability, and high sensitivity to cryopreservation. In this study, we established a feeder-free culture system that selectively activated and expanded NK cells directly from peripheral blood mononuclear cells (PBMCs) without prior depletion of T or B cells. The expanded NK cells exhibited increased CD16 expression compared with naïve NK cells and demonstrated enhanced antitumor activity through antibody-dependent cellular cytotoxicity (ADCC) when combined with trastuzumab. To overcome limitations in long-term storage, NK cells were cryopreserved using a formulation containing 5% dimethyl sulfoxide (DMSO) supplemented with sugars and albumin. Importantly, the *in vivo* antitumor efficacy of cryopreserved NK cells was found to be comparable to that of freshly expanded NK cells. These findings show that feeder-free expanded NK cells possess potent ADCC activity and that optimized cryopreservation strategies can preserve therapeutic efficacy, supporting their clinical applicability and scalability as not only off-the-shelf allogeneic products but also personalized autologous NK cell therapies.

## Introduction

In the field of cell therapy, advances in CAR-T cell therapy have led to major breakthroughs in cancer immunotherapy. Tumor-infiltrating lymphocyte (TIL) therapy and TCR-based T cell therapies have also demonstrated promising efficacy, with several products receiving FDA approval [1-6]. However, T cell–based therapies are associated with serious adverse effects, including cytokine release syndrome (CRS), immune effector cell–associated neurotoxicity syndrome (ICANS), and hemophagocytic lymphohistiocytosis (HLH). Moreover, in the case of allogeneic administration, there remain risks of prolonged cytopenia and graft-versus-host disease (GvHD) [7-10].

In contrast, NK cell–based therapies are associated with minimal adverse effects and are therefore considered a safer alternative [11]. NK cell therapeutics can be developed in both autologous and allogeneic settings. In particular, allogeneic NK cells can be derived and expanded from diverse sources, including peripheral blood (PB), cord blood (CB), bone marrow (BM), and human induced pluripotent stem cells (iPSCs) [12]. However, compared with autologous NK cells, allogeneic NK cells generally exhibit shorter persistence *in vivo*, necessitating repeated infusions [13, 14]. Moreover, donor variability may lead to mismatches between donor and recipient, potentially resulting in unpredictable responses, such as diminished efficacy or excessive immune activation [15, 16].

Autologous NK cell therapy utilizes NK cells derived from a patient’s own peripheral blood. Typically, NK cells are isolated, expanded, and activated *ex vivo*, and then reinfused into the patient to enhance antitumor immunity. However, NK cells constitute only about 5–10% of peripheral blood mononuclear cells (PBMCs), making them more difficult to obtain compared with T cells [17]. Consequently, various expansion protocols have been developed to generate sufficient numbers and purity of NK cells while preserving their functional activity [18]. NK cell expansion methods are broadly categorized into feeder cell–based and feeder-free approaches. Feeder cell–based expansion achieves strong proliferation and activation but often relies on leukemic cancer cell lines, raising safety concerns and regulatory restrictions. In contrast, cytokine-based feeder-free expansion is relatively safer and more amenable to GMP-compliant manufacturing, although it is generally limited by lower expansion efficiency. Thus, establishing efficient feeder-free platforms that ensure both safety and scalability remains a critical challenge in NK cell therapy development.

In this study, we established a feeder-free culture system that selectively activated and expanded NK cells directly from PBMCs without prior T- or B-cell depletion. The antitumor activity of the expanded NK cells was evaluated by analyzing their antibody-dependent cellular cytotoxicity (ADCC) function in combination with trastuzumab. Furthermore, for the future development of off-the-shelf cell therapies, the establishment of effective cryopreservation strategies enabling long-term storage is essential. However, NK cells are highly sensitive to the freeze–thaw process, which has been reported to cause diminished cytotoxic activity, reduced viability, and functional impairment. Recent studies have suggested that cryopreservation media containing 5%dimethyl sulfoxide (DMSO) may be advantageous for preserving NK cells [19]. Accordingly, in this study, NK cells were cryopreserved using a formulation consisting of 5% DMSO supplemented with sugars and albumin, and their *in vivo* antitumor efficacy was directly compared with that of freshly expanded NK cells.

## Materials and Methods

### Cancer Cell Culture

Human breast cancer cell lines, MDA-MB-453, BT-474 and SKBR-3 were cultured in RPMI-1640 supplemented with 10% heat-inactivated fetal bovine serum (FBS), 100 U/ml penicillin and 0.1 mg/ml streptomycin (Invitrogen, USA). The human gastric cancer cell lines NCI-N87 and the leukemia cell line K562 were maintained under the same culture conditions. All cell lines were purchased from Korean Cell Line Bank (KCLB, Republic of Korea).

### Antibodies

The anti-human HER-2 antibody was purchased from Roche (Switzerland), and the human IgG1 isotype control was purchased from BioXCell (USA). Antibodies used for flow cytometry analysis are listed in Table 1. Cells were stained with antibodies in the dark at 4°C for 20min. Stained cells were analyzed using CytoFLEX flow cytometer (Beckman Coulter, USA), and data was analyzed using FlowJo version 10.1 software (Treestar Inc., USA).

### Expanded NK Cells

The study using human blood was approved by the Institutional Review Board of CHA Bundang Medical Center, CHA University (IRB no. 2012–175). Expanded NK cells were cultured *ex vivo* according to previously described protocols [20]. In brief, PBMC were seeded on a γ-globulin (Greencross, Republic of Korea) and anti-NKp46 (R&D Systems, USA)-coated flask and cultured in Alys505NK serum-free medium (CSTI, Japan) supplemented with 1,000 IU/ml recombinant human IL-2 (Novartis, Switzerland), 50 ng/ml recombinant human IL-18 (BioLegend, USA), and 5% heat-inactivated autologous plasma. Recombinant human IL-18 was added at the initiation of culture and again on day 5. Fresh culture medium was added every 1 to 3days depending on the cell density (2 × 10^6^ cells/ml). On Day 6, the cells were transferred to a culture bag (NIPRO, Japan) and cultured for 14days. The expanded NK cells were cryopreserved in a formulation containing 5% dimethyl sulfoxide (DMSO; BioLife Solutions, USA), albumin (Greencross, Republic of Korea), and pentastarch (Jeil Pharm, Republic of Korea).

### CD107a Degranulation Assay

Expanded NK cells were cocultured with target cells (BT-474, MDA-MB-453, SKBR-3, and NCI-N87) at an Effector:Target (E:T) ratio of 5:1 and anti-CD107a-PE (eBiosciences) an trastuzumab for 4h. After coculturing, cells were stained with anti-CD3-FITC and anti-CD56-APC antibodies. CD107a expressed on CD3^−^CD56^+^ NK cells was analyzed using a CytoFLEX flow cytometer, and data were analyzed using FlowJo version 10.1 software.

### Cell Cytotoxicity

NK cell cytotoxicity against cancer cells was evaluated using carboxyfluorescein succinimidyl ester/7-aminoactinomycin D (CFSE/7-AAD) flow cytometry-based assay. Target tumor cells were labeled with CFSE (Thermo Fisher Scientific, USA) at 37°C for 10 min, and then co-incubated with NK cells at various E:T ratios for 4 h at 37°C. After incubation, cells were resuspended in PBS containing 7-AAD (Thermo Fisher Scientific), and cytotoxicity was assessed by measuring the percentage of CFSE^+^7-AAD^+^ target cells using a CytoFLEX flow cytometer and FlowJo software. For ADCC activity analysis, NK cells were co-cultured with CFSE-labeled target cells in the presence of trastuzumab. Trastuzumab was applied in a concentration-dependent manner to evaluate its combinatorial effect with NK cells.

### ELISA Analysis

To assess ADCC activity, effector and target cells were co-cultured for 24 h at the same E:T ratio used in the cytotoxicity assay, and the collected supernatants were subjected to ELISA analysis. IFN-γ in cell culture supernatants was measured using Human IFN-γ ELISA Set (R&D Systems) according to the manufacturer’s protocol.

### *In Vivo* Xenograft Gastric Cancer Model

Gastric tumor modeling was established in 4- to 5-week-old female NSG mice (JAbio, Republic of Korea). NCI-N87 cells (5×10^6^ cells) were subcutaneously inoculated in the right flank, tumor volume was calculated using the formula: volume=(width^2^ ×length)/2. Once tumors reached a volume of approximately 80–100 mm^3^, the mice were randomized into six treatment groups: (1) negative control, (2) trastuzumab (2 mg/kg), (3) NK cells (fresh),(4) NK cells (frozen), (5) NK cells (fresh) + trastuzumab (2 mg/kg), and (6) NK cells (frozen) + trastuzumab (2 mg/kg). NK cells were administered intravenously at a 1×10^7^ cells/mouse in both the NK cell monotherapy and combination groups. Trastuzumab was co-administered simultaneously with NK cells. Tumor volume was monitored up to day 39 following treatment initiation. All animal procedures were reviewed and approved by the Institutional Animal Care and Use Committee of CHA University (IACUC no. 1601114), and conducted in accordance with institutional and national guidelines.

### Statistical Analysis

Data are presented as mean ± standard deviation (SD). Statistical significance was determined by *t*-test between two groups, and by one-way or two-way ANOVA followed by Tukey’s post-hoc test for multiple group comparisons. *P ≤ 0.05, **P ≤ 0.01, ***P ≤ 0.001, ****P ≤ 0.0001 were considered statistically significant. Statistical analysis is performed with GraphPad Prism Statistical (GraphPad, USA).

## Results

### Characterization of *Ex Vivo* Expanded NK Cells for 14Days

**Fig. 1:**
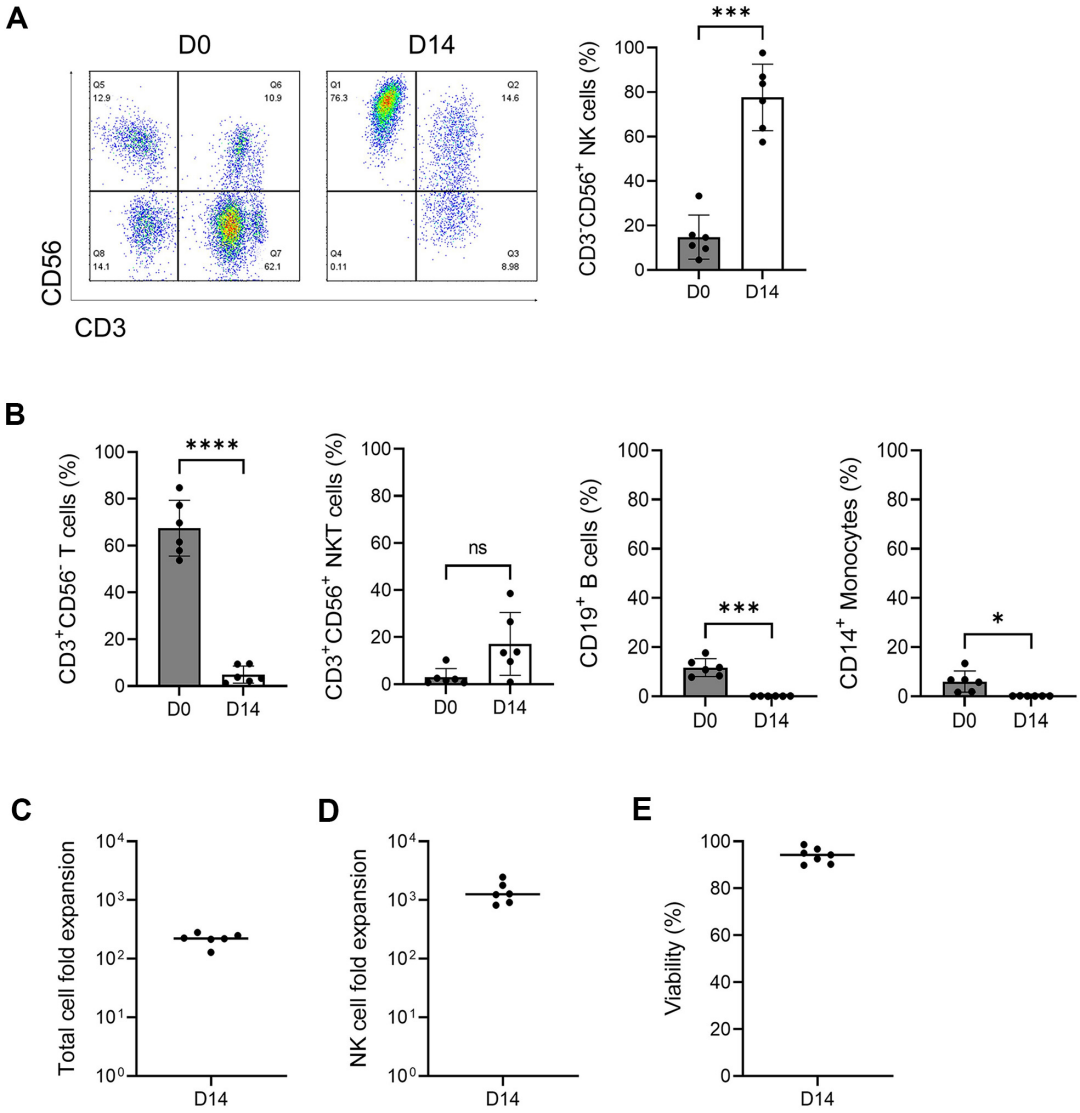
Phenotypic analysis of PBMCs and expanded NK cells. The phenotype of PBMC (D0) and activated NK cells cultured for 14 days (D14) were analyzed by flow cytometry. (**A**) Distribution of CD3^-^CD56^+^ NK cells in PBMC (D0) and after culture D14. (**B**) The distribution of CD3^+^CD56^+^ NKT cells and CD3^+^CD56^-^ T cells and CD19^+^ B cells in PBMC (D0) and activated NK cells (D14). The distribution of CD14+ Monocytes in PBMC and activated cells (D14). (**C**) Fold expansion of total cells at D14 of the culture. Line at mean (n=6). (**D**) Fold expansion of NK cell population (CD3^-^CD56^+^) at D14 of the culture. Line at mean (n=6). (**E**) Cell viability of immune cells at D14 of culture. Line at mean (n=6). Statistical analysis was performed using a paired *t*-test (*P < 0.05, **P < 0.01, ***P < 0.001, and ****P < 0.0001).

The phenotype of cells isolated from the blood of six independent donors, using PBMCs from day 0 (D0), was analyzed following a two-week culture with antibodies and cytokines. A comparison of the proportions of CD3^-^CD56^+^ NK cells between D0 and D14 revealed a significant increase in NK cells, from 14.8% (±9.9%) at D0 to 77.6% (±15.0%) at D14 (Fig. 1A). In contrast, the proportion of CD3^+^CD56^-^ T cells decreased from 75.8%(±11.3%) at D0 to 11.5% (±13.2%) at D14, and both B cells and monocytes also showed a decrease after culture, with proportions of 0.1% (±0.1%) and 0.1% (±0.0%), respectively. Although the proportion of CD3^+^CD56^+^ NKT cells increased from 2.9% (±3.7%) at D0 to 17.1% (±13.3%) at D14, this change did not reach statistical significance (Fig. 1B).

In the feeder-free culture system used in this study, the expansion rate of the total cell number at D14 was approximately 219-fold (range: 128–279) (Fig. 1C). The expansion potential of NK cells reached 1,412-fold (range: 818–2,462) (Fig. 1D), and the mean cell viability at the time of harvest was 94.6% (±3.0%) (Fig. 1E). These findings indicate that the cultured population consisted predominantly of expanded NK cells.

**Fig. 2:**
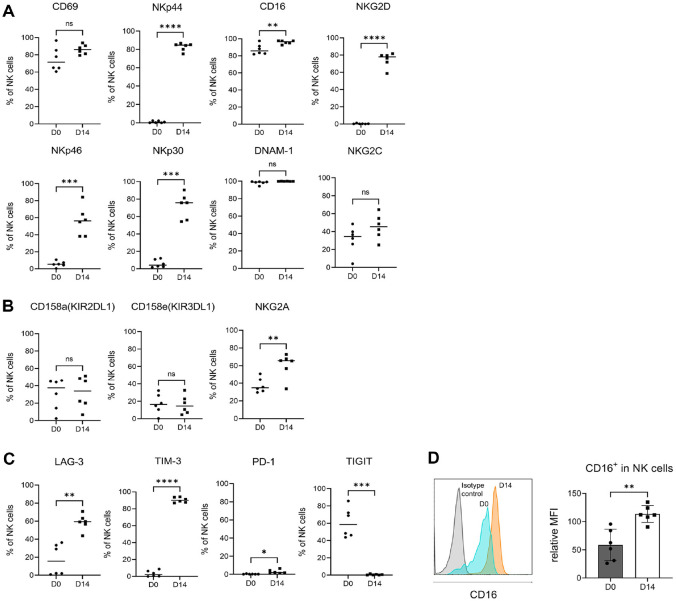
Expression of activating, inhibitory, and exhaustion markers on NK cells before and after expansion. (**A-C**) Phenotypic analysis of NK cells before and after expansion. Surface expression of (**A**) activating receptors, (**B**) inhibitory receptors, (**C**) exhaustion markers of NK cells from 6 different donors was analyzed using flow cytometry before (D0) and after expansion (D14). (**D**) Expression of CD16 on NK cells was analyzed by flow cytometry. The histograms on the left show CD16 expression in NK cells from D0 and expanded NK cells (D14) compared with the isotype control. The relative mean fluorescence intensity (MFI), calculated by dividing the MFI of CD16 expression by that of the isotype control, is shown on the right. Statistical analysis was performed using a paired *t*-test (*P < 0.05, **P < 0.01, ***P < 0.001, and ****P < 0.0001).

To compare expanded NK cells with PBMCs before culture, we assessed the expression of various NK cell receptors using flow cytometry. The expression of activating receptors, including NKG2D and natural cytotoxicity receptors (NCRs) such as NKp30, NKp44, and NKp46, was significantly increased after culture compared to before culture (D0), and the expression of CD16 also showed a statistically significant moderate increase. Additionally, the expression of CD69 and NKG2C was found to be elevated (Fig. 2A). Among inhibitory receptors, CD158a (KIR2DL1) and CD158e (KIR3DL1) showed a trend toward a slight decrease after culture, although this was not statistically significant. However, NKG2A expression was found to be upregulated following culture (Fig. 2B). Regarding exhaustion markers, the expression of LAG-3 and TIM-3 increased after culture, while TIGIT showed almost no expression after culture (Fig. 2C). The expression of CD16 plays a crucial role in mediating ADCC. The expression of CD16 on NK cells was compared before and after culture. The mean fluorescence intensity (MFI) of CD16 expression was measured relative to the isotype control, and the ratio of the MFI of CD16 to the MFI of the isotype control was calculated as the relative MFI (rMFI). The rMFI of CD16 at D0 was 58.6 (±28.0), while the rMFI of CD16 at D14 was 113.7 (±14.9), showing a trend toward an increase in CD16 expression intensity after culture (Fig. 2D).

### Cytotoxicity of Expanded NK Cells against HER2-Positive Cancer Cell Lines

**Fig. 3:**
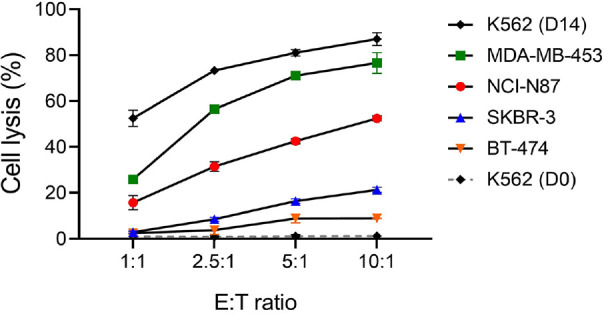
Cytotoxic activity of expanded NK cells against HER2-positive cancer cell lines. NK cell-mediated cytotoxicity was assessed using a 7-AAD assay with E:T ratios of 1:1, 2.5:1, 5:1, and 10:1. K562 cells were used as a positive control for NK sensitivity. HER2-positive tumor cell lines included MDA-MB-453 and BT-474 (breast cancer), SKBR-3 (breast cancer), and NCI-N87 (gastric cancer). Expanded NK cells showed dose-dependent cytotoxicity, with the highest lysis observed in K562 and MDA-MB-453 cells. Data are presented as mean ± SD from two independent donor experiments.

We evaluated the cytotoxic activity of expanded NK cells against various HER2-positive cancer cell lines. K562 cells, known to be highly sensitive to NK cell-mediated lysis, were used as a positive control. PBMCs (D0) exhibited minimal cytotoxic activity, with cell lysis rates remaining below 3% even at the highest effector-to-target (E:T) ratio of 10:1. In contrast, NK cells expanded for 14 days showed significantly enhanced cytotoxic function, achieving a lysis rate of 87.0% (± 2.8%) against K562 cells at the same E:T ratio. Among the HER2-positive tumor cell lines, MDA-MB-453 exhibited the highest sensitivity to expanded NK cell-mediated cytotoxicity, with cell lysis reaching 76.6% (± 4.5%) at the highest E:T ratio. NCI-N87 showed moderate susceptibility, whereas SKBR-3 and BT-474 exhibited relatively low sensitivity, with lysis rates not exceeding 25% even at the maximum E:T ratio tested (Fig. 3).

### Functional Assay of Expanded NK Cells and Evaluation of ADCC Activity

**Fig. 4:**
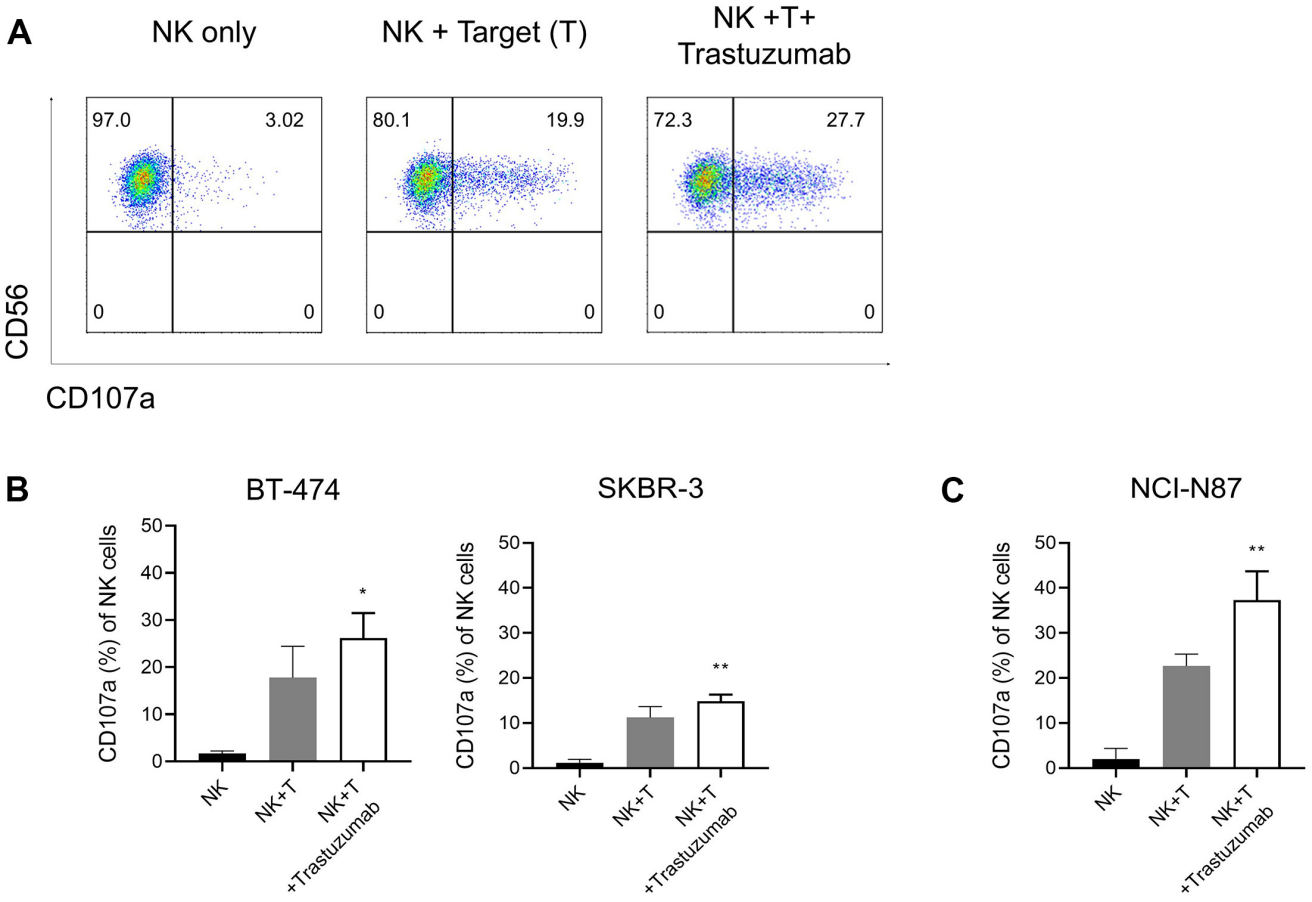
*In vitro* ADCC activity of expanded NK cells assessed by CD107a expression. (**A**) Representative flow cytometry plots showing CD107a surface expression on gated CD3-CD56^+^ NK cells in the absence of target cells (NK only), in the presence of HER2-positive target cells (NK + Target), and with trastuzumab treatment (NK + T + Trastuzumab). (**B–C**) Quantification of CD107a^+^ NK cells following co-culture with HER2-positive cancer cell lines in the absence or presence of trastuzumab. (**B**) BT-474 and SKBR-3, (**C**) NCI-N87. Trastuzumab treatment significantly increased the proportion of CD107a^+^ NK cells, indicating enhanced degranulation and antibody-dependent cellular cytotoxicity (ADCC). Data are presented as mean ± SD. Statistical significance was determined using one-way ANOVA with Tukey’s post hoc test (*P < 0.05, **P < 0.01, ***P < 0.001).

To evaluate the ADCC activity of expanded NK cells, a CD107a degranulation assay was performed using HER2-positive tumor cell lines, including the breast cancer cell lines BT-474 and SKBR-3, and the gastric cancer cell line NCI-N87. In the absence of target cells, NK cells exhibited minimal CD107a expression. However, co-culture with target cells induced CD107a expression, which was further enhanced by trastuzumab treatment, confirming the increased ADCC effect (Fig. 4A). In BT-474 cells, treatment with NK cells alone resulted in 17.8%(± 6.6%) cytotoxicity, which increased to 26.2% (± 5.3%) with trastuzumab co-treatment. In SKBR-3 cells, cytotoxicity increased from 11.2% (± 2.4%) with NK cells alone to 14.9% (± 1.4%) with trastuzumab (Fig. 4B). In the gastric cancer cell line NCI-N87, CD107a expression by NK cells significantly increased from 22.7% (± 2.6%) to 37.3% (± 6.4%) upon trastuzumab co-treatment, confirming a synergistic effect mediated by ADCC (Fig. 4C).

**Fig. 5:**
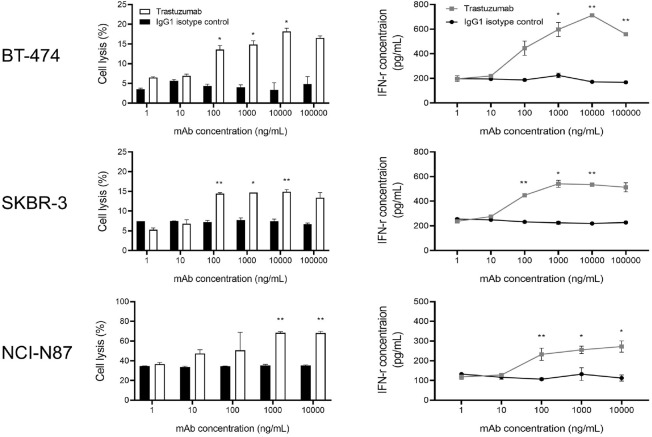
Dose-dependent enhancement of cytotoxicity and IFN-γ secretion by trastuzumab across HER2-positive cancer cell lines. Expanded NK cells were co-cultured with HER2-positive tumor cell lines—BT-474, SKBR-3, and NCIN87— in the presence of trastuzumab or an IgG1 isotype control at concentrations ranging from 1 to 10,000 ng/ml. **Left panels:** NK cell-mediated cytotoxicity was assessed by measuring the percentage of target cell lysis. Trastuzumab treatment led to a dose-dependent increase in cytotoxic activity. **Right panels:** After 24 h of co-culture under the same conditions, supernatants were collected and IFN-γ levels secreted by NK cells were quantified using ELISA. Trastuzumab significantly enhanced IFN-γ secretion compared to the isotype control, and this effect was dose-dependent. Data represent the mean ± standard deviation (SD) from independent experiments using NK cells derived from at least two different donors. Statistical significance between trastuzumab and IgG1 control at each concentration was determined using an unpaired *t*-test. (*P < 0.05, **P < 0.01, ***P < 0.001).

To evaluate the ADCC activity of trastuzumab, cell lysis and IFN-γ secretion were measured in HER2-positive cancer cell lines following co-culture with NK cells. At the same antibody concentration, all tested HER2-positive cell lines showed significantly enhanced cytotoxicity and IFN-γ production in the trastuzumab-treated groups compared to the IgG1 isotype control. While trastuzumab induced a dose-dependent increase in NK cell-mediated cytotoxicity, no significant changes were observed in the IgG1-treated controls, regardless of antibody concentration. At 1,000 ng/ml, BT-474 cells showed an increase in cell lysis from 4.1% (± 0.6%) in the control group to 14.9% (± 1.0%) in the trastuzumab group, representing a 3.6-fold enhancement. IFN-γ secretion also increased from 224.6 pg/ml (± 18.6) to 597.7 pg/ml (± 58.8), indicating a 2.6-fold elevation. Similarly, SKBR-3 cells exhibited an increase in cell lysis from 4.1% (± 0.6%) to 14.7% and IFN-γ secretion from 225.1 pg/ml to 541.4 pg/ml, demonstrating approximately 3.5-fold and 2.6-fold increases, respectively. In the NCI-N87 gastric cancer cell line, trastuzumab treatment at 1,000 ng/ml significantly enhanced cytotoxicity, increasing cell lysis from 35.2%(± 1.3%) to 68.3% (± 1.3%), confirming a potent ADCC effect with an approximately 1.9-fold increase (Fig. 5).

### Comparative Anti-Tumor Efficacy of Fresh and Cryopreserved NK Cells in Combination with Trastuzumab *In Vivo*

**Fig. 6:**
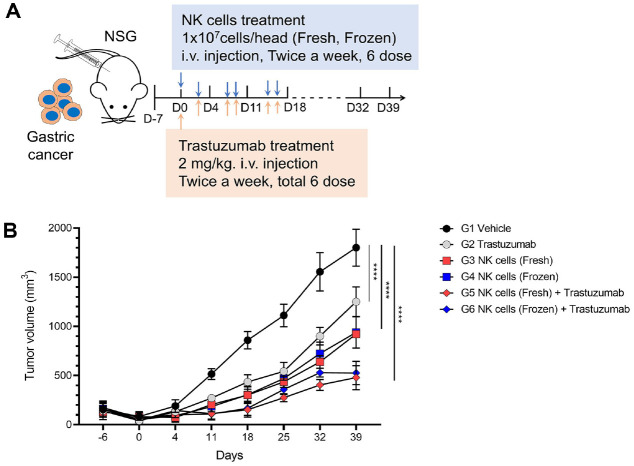
*In vivo* comparison of fresh and cryopreserved NK cell formulations in trastuzumab combination therapy for HER2-positive gastric cancer. (**A**) Evaluation of ADCC efficacy based on NK cell formulation in a gastric cancer model. Mice of the same age were subcutaneously injected with 5×10^6^ NCI-N87 cells. A high dose (1×10^7^) of expanded NK cells was administered intravenously in either fresh or cryopreserved form. The cryopreserved formulation included 5% DMSO along with albumin and pentastarch. In the combination group, trastuzumab was added to the NK cell treatment and administered concurrently with NK cells. (**B**) Tumor volume was monitored for up to 39 days. Statistical significance was determined using one-way ANOVA followed by Tukey’s multiple comparisons test. ****p < 0.0001 versus G1 Vehicle.

To evaluate *in vivo* ADCC activity, a subcutaneous xenograft mouse model was established using HER2-positive NCI-N87 gastric cancer cells. This study also compared the anti-tumor efficacy between fresh and cryopreserved NK cell formulations (Fig. 6A). During the treatment period, all treatment groups showed significantly slower tumor growth compared to the vehicle group. After stopping treatment, the trastuzumab monotherapy group showed the most rapid tumor regrowth, followed by the NK cell monotherapy group. In contrast, the combination groups (NK cells + trastuzumab) maintained a sustained tumor-suppressive effect even after treatment withdrawal. Although the fresh NK cell formulation demonstrated slightly better efficacy, the cryopreserved formulation also showed comparable anti-tumor activity (Fig. 6B).

The tumor growth was significantly inhibited in the NK cell monotherapy groups compared to the vehicle group, with inhibition rates of 49% for the fresh formulation and 48% for the frozen formulation (*P* < 0.001). Notably, the combination groups showed greater tumor suppression, with inhibition rates of 73% and 71% for the fresh and frozen formulations, respectively (*P* < 0.001), demonstrating superior efficacy compared to NK cell monotherapy. In contrast, the trastuzumab monotherapy group showed only a 31% reduction in tumor growth compared to the vehicle group (*P* < 0.001), indicating markedly lower efficacy.

## Discussion

In this study, we isolated peripheral blood mononuclear cells (PBMCs) from blood and produced expanded NK cells over 14 days using a feeder-free method with antibodies and cytokines. NK cells obtained from six donors were analyzed before (D0) and after (D14) culture to examine changes in NK receptor expression, and ADCC activity targeting HER2-positive cancer cells was evaluated through *in vitro* cell cytotoxicity assays and an *in vivo* xenograft mouse model.

Cancer immunotherapy is attracting attention as a promising treatment strategy in various types of cancer. The anti-tumor activity of NK cells has already been demonstrated in animal models. However, clinical application of this strategy in humans has only recently begun to be explored in earnest. Unlike T cells, NK cells carry a minimal risk of inducing GvHD, making them a clinically flexible platform for cell-based immunotherapy. This property allows NK cells to be effectively applied in both allogeneic therapies using PBMCs from healthy donors and autologous therapies using PBMCs derived from the patient [21, 22]. This approach allows for the generation of NK cells from both healthy donor-derived PBMCs for allogeneic applications and patient-derived PBMCs for autologous use. Allogeneic NK cells exhibit relatively strong antitumor activity; however, it has been reported that their persistence *in vivo* is limited due to recognition and elimination by the host immune system, often resulting in only short-term efficacy [23]. In contrast, autologous NK cells have the advantages of a lower risk of immune rejection, suitability for repeated administration, and potential as personalized therapeutics [24, 25].

In this study, we demonstrated that NK cells can be selectively activated and efficiently expanded from heterogeneous PBMCs without requiring CD3 depletion or CD56^+^ cell sorting. After 14 days of culture using cells from six different donors, the total cell number increased by approximately 219-fold, while NK cells specifically expanded by about 1,412-fold.

We compared the expression of NK receptors before and after expansion. Major activating receptors, including NKp44, CD16, NKG2D, NKp46, and NKp30, were significantly upregulated, while among inhibitory receptors, the expression of NKG2A was increased. exhaustion-related receptors LAG-3 and TIM-3 were increased, while TIGIT expression was decreased. LAG-3 and TIM-3 are known as exhaustion markers for T cells and NK cells, and TIGIT functions as an inhibitory receptor that binds to CD155 (PVR) and suppresses cytotoxicity and cytokine secretion [26-29]. These results suggest that although the NK cells were in a partially exhausted state, the reduction of TIGIT expression may minimize inhibitory signaling, while the regulated expression of LAG-3 and TIM-3 may reflect a complex immune regulatory state that helps maintain anti-tumor activity. Furthermore, the expression of CD16, a key mediator of ADCC activity, was analyzed based on mean fluorescence intensity (MFI), and a significant increase in CD16 expression was observed after culture. This supports the potential enhancement of ADCC function in *ex vivo* expanded NK cells.

We assessed the cytotoxic activity of expanded NK cells by comparing K562 cell lysis before and after expansion. Despite using a high E:T ratio, PBMCs (D0) showed low cytotoxicity. In contrast, expanded NK cells showed robust cytotoxicity, with over 55% cell lysis observed even at a 1:1 E:T ratio. When tested against HER2-positive cancer cell lines, the degree of responsiveness to NK cells varied depending on the cell line, and cell lysis increased proportionally with the E:T ratio.

In addition, the ADCC activity of expanded NK cells against HER2-positive cancer cell lines was evaluated under varying concentrations of trastuzumab, based on both cell lysis and IFN-γ secretion. In all three HER2-positive cell lines tested, the trastuzumab-treated groups showed significantly higher levels of cell lysis and IFN-γ secretion compared to the IgG1 isotype control, confirming that the combination of expanded NK cells with trastuzumab induces a synergistic ADCC effect.

To effectively use NK cells as a therapeutic agent, a cryopreservation technology is essential. However, NK cells are highly sensitive to freeze-thaw processes, and concerns have been continuously raised regarding functional impairments such as reduced cytotoxicity and decreased viability. In this study, we evaluated the *in vivo* ADCC activity of NK cells in a HER2-positive gastric cancer model, comparing the anti-tumor efficacy of fresh and cryopreserved (frozen) NK cell formulations. As a result, combination treatment with trastuzumab showed superior tumor growth inhibition compared to NK cell monotherapy. Moreover, there was no significant difference in anti-tumor efficacy between the fresh and frozen formulations, indicating comparable therapeutic performance. In contrast, trastuzumab monotherapy resulted in only a 31% reduction in tumor growth, demonstrating relatively limited efficacy.

## Conclusion

In this study, we confirmed that NK cells can be effectively expanded from PBMCs using a feeder-free system. The expanded NK cells exhibited enhanced ADCC activity against HER2-positive cancer cells when combined with trastuzumab, and cryopreserved formulations retained antitumor efficacy comparable to fresh cells. These findings suggest that a single blood collection may yield multiple therapeutic doses and highlight the potential of activated NK cells as a versatile platform for developing clinical strategies to overcome refractory cancers.

## Figures and Tables

**Table 1:** Antibodies used for the analysis of expanded NK cells.

Material	Clone	Vender	Catalog #
CD314 (NKG2D)	1D11	eBioscience	12-5878-42
CD158e1 (KIR3DL1)	DX9	Biolegend	312706
CD366 (TIM3)	F38-2E2	eBioscience	11-3109-42
CD158 (KIR2DL1/S1/S3/S5)	HP-MA4	Biolegend	339504
CD3, FITC	UCHT1	eBioscience	11-0038-42
CD19	HIB19	eBioscience	12-0199-42
CD336 (NKp44)	P44-8	Biolegend	325108
CD226 (DNAM-1)	11A8	Biolegend	338306
CD16	CB16	eBioscience	12-0168-42
CD14	61D3	eBioscience	12-0149-42
CD335 (NKp46)	9E2	Biolegend	331908
CD69	FN50	Biolegend	310926
CD279 (PD-1)	EH12.2H7	Biolegend	329914
CD337 (NKp30)	P30-15	Biolegend	325216
NKG2C (CD159c)	S19005E	Biolegend	375020
NKG2A (CD159a)	S19004C	Biolegend	375104
LAG-3 (CD223)	T47-530	BD	565616
TIGIT (VSTM3)	A15153G	Biolegend	372718
CD56 (NCAM)	CMSSB	eBioscience	17-0567-42
FTIC	P3.6.2.8.1	eBioscience	11-4714-42
PE	P3.6.2.8.1	eBioscience	12-4714-42
APC	P3.6.2.8.1	eBioscience	17-4714-82
PC.5.5	P3.6.2.8.1	eBioscience	45-4714-82
CD3, eFluor 450	UCHT1	eBioscience	48-0038-42
